# Long non-coding RNA deep sequencing reveals the role of macrophage in liver disorders

**DOI:** 10.18632/oncotarget.23154

**Published:** 2017-12-12

**Authors:** Zhang Lin, Hao Changfu, Zhao Fengling, Guo Wei, Bao Lei, Li Yiping, Zhang Miao, Yue Zhongzheng, Zhao Youliang, Duan Shuyin, Yao Wu

**Affiliations:** ^1^ Department of Occupational and Environmental Health, School of Public Health, Zhengzhou University, Zhengzhou 450001, China; ^2^ Center for Reproductive Medicine, Shandong Provincial Hospital Affiliated to Shandong University, Jinan 250001, China; ^3^ Key Laboratory of Reproductive Endocrinology, Shandong University, Ministry of Education, National Research Center for Assisted Reproductive Technology and Reproductive Genetics, Jinan 250001, China; ^4^ Department of Occupational Disease, Henan Provincial Institute of Occupational Health, Zhengzhou 450052, China

**Keywords:** macrophage, liver disorder, lncRNA profiling, cancer, biomarker

## Abstract

Liver disorders such as hepatitis, cirrhosis and hepatocellular carcinoma are a series of the most life threatening diseases along with extensive inflammatory cellular infiltrations. Macrophage has been proved to be key regulators and initiators of inflammation, and long non-coding RNAs (lncRNAs) are recommended to play critical roles in the occurrence and development of a variety of diseases. To uncover the role of macrophage in liver disorders via lncRNA sequencing method, we first applied a lncRNA classification pipeline to identify 1247 lncRNAs represented on the Affymetrix Mouse Genome 430/430A 2.0 array. We then analyzed the lncRNA expression patterns in a set of previously published gene expression profiles of silica particle exposed macrophages and liver respectively, and identified and validated sets of differentially expressed lncRNAs shared by macrophages and liver. The functional enrichment analysis of these lncRNAs was processed on the basis of their expression signatures, three aspects including *cis*, *trans* and co-acting proteins were proposed. This is the first time to correlate macrophage with liver disorders via co-expressed lncRNAs. Our findings indicated that roles of macrophage in liver disorders were double-edged, the differentially expressed lncRNAs and their corresponding regulatory genes or proteins may serve as potential diagnostic biomarkers and therapeutic targets.

## INTRODUCTION

Long term or acute exposure to silica dust may cause liver disorders, and a variety of pathological changes such as inflammatory cellular infiltration, immune imbalance, and fibrosis may be induced at the early stage [1]. Consequently, cirrhosis and hepatocellular carcinoma, which are of serious life threatening and cannot be reversed once occurred [2, 3], also come into being on the basis of such changes in the end. Up to now, liver diseases that associated with dust exposure have caused a significant loss in human resources, material resources and financial resources, what is worse, the number of patients has been snowballing in recent years [4]. Since there is still no effective biomarkers for early stage diagnosis of those diseases, liver cancer, for example, one of the most common malignancies worldwide, hold a great proportion of cancer-related deaths [5]. Mechanistically, plenty of mononuclear phagocytes primarily macrophages would aggregate to scavenge foreign bodies under inflammatory conditions. As suggested by previous studies, the phagocytosis was the first step in humoral immune responses and was accompanied by releasing of numerous inflammatory factors and cytokines [6]. And it was also essential in the emergence and development of many kinds of diseases, including pulmonary fibrosis [7], tuberculosis [8], silicosis [9], hepatitis [10] and liver cancer [11]. In addition, macrophages that distributed in other tissues or organisms can also be transported to liver via blood circulation [12]. Therefore, it would be of great significance to figure out the role of macrophage in liver disorders, which may also contribute to identifying biomarkers and novel targets for disease early diagnosis.

To date, considerable attentions have been drawn on long non-coding RNAs (lncRNAs) which have been identified in many cancers [13–15]. lncRNAs are non-protein-coding transcripts longer than 200 nucleotides involved in numerous critical biological processes such as X chromosome silencing, genomic imprinting, chromosome modification, transcriptional activation, transcriptional interference, and nuclear transport [16]. The functional mechanisms are diversely distributed in patterns of scaffolds, decoys, guides or signals, and shown as *cis* or *trans* regulation of transcription and post-transcription, antisense interference, epigenetic modification [17]. However, functions of the majority of lncRNAs have not been fully explored when compared with non-coding RNAs less than 200 nucleotides, especially microRNAs, approximately 19 ∼25 nucleotides, which were well studied and found to be critical factors in daily physiological activities and diseases [18–20]. With advancements in the next generation genome sequencing technologies, more and more evidence suggests that lncRNAs also play important roles in keeping the normal physiological metabolism, and the aberrant expression of lncRNAs is associated with functional disorders [21]. In liver, as indicated by Lanaya Hanane and colleagues, lncRNAs participate in macrophage inflammatory response and immune imbalance locally or systematically, promoting fibroblasts proliferation and trans-differentiation, tissue fibrosis and cancer metastasis [22]. The expression of lncRNAs also reflects disease progression and can potentially serve as predictors in disease diagnosis and prognosis. For example, metastasis-associated lung adenocarcinoma transcript 1 (MALAT1), the earliest found cancer-related lncRNA, could promote cancer cells growth in non-small cell lung cancer (NSCLC). However, the metastatic ability attenuated after inhibiting the expression of MALAT1 [23]. Another well-studied lncRNA, X-inactive specific transcript (XIST), could directly interact with miR-92b and repress each other, besides, XIST could inhibit hepatocellular carcinoma cell proliferation and metastasis [24]. In spite of critical roles of lncRNAs and macrophages in liver diseases, it has not been investigated that whether aberrantly expressed lncRNAs shared by macrophage and liver can be used to identify roles of macrophage in liver diseases. Coincidently, studies published previously have provided available profiling data, and the microarray datasets can be achieved from the Gene Expression Omnibus (GEO). Due to many lncRNA-specific probes are represented on these commercial arrays, we can use these existing data to deep sequence expression signatures of lncRNAs.

In this study, we aimed at profiling the lncRNA expression signatures in silica exposed macrophages and liver by analyzing a cohort of previously published microarray data sets that achieved from the GEO. The identified differentially expressed lncRNAs were validated using real time PCR method. Our findings provide novel information on lncRNA expression profiles that may help to elucidate the role of macrophage in liver diseases, as well as identify potential diagnostic biomarkers and signaling pathways.

## RESULTS

### Data sets characteristics

**Figure 1:**
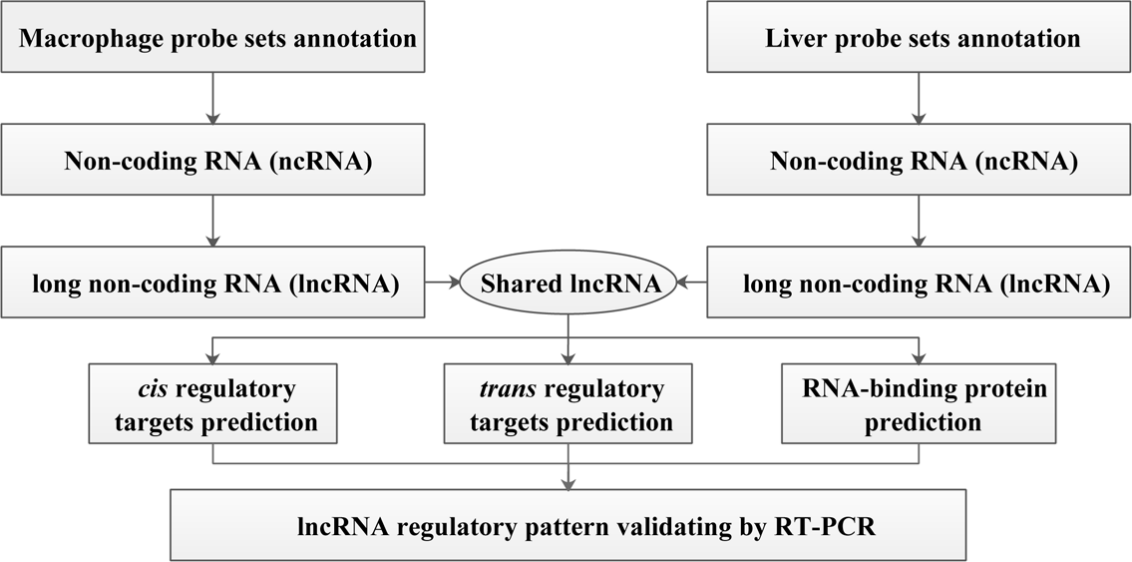
Schematic overview of the workflow

The gene expression data of macrophage and liver along with their corresponding controls were included in this study: GSE13005 and GSE30861. The gene chip GSE13005 contained 21 samples, among which 18 samples were exposed to silica particles, while three samples in the control group were silica particle non-exposed. GSE30861 included 35 samples, of theses, 30 samples in the silica particle exposure group as well as five non-stimulated controls. In the quality control process, seven samples in GSE13005 and 17 samples in GSE30861 which fell well outside the control limits for both NUSE metrics and the borderline on the RLE IQR plot were removed. Consequently, 14 samples (case vs. control: 11 vs. 3) in GSE13005 and 18 samples (case vs. control: 14 vs. 4) in GSE30861 were involved in the further data mining procedures in this study, and the entire experiment work flow was summarized in Figure 1.

### lncRNA expression profiles on affymetrix mouse genome 430A/430 2.0 arrays

**Figure 2:**
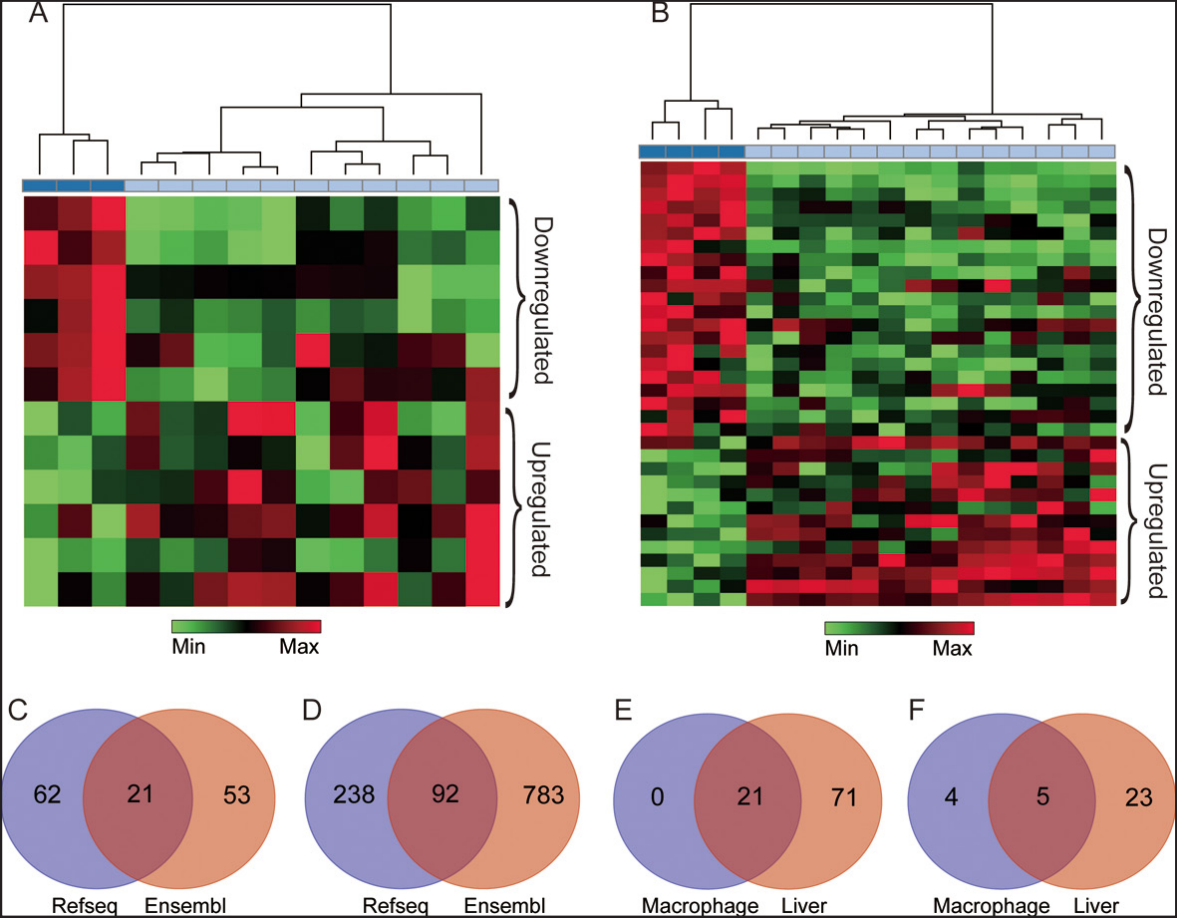
lncRNAs extracted from silica particle exposed macrophages and liver The results of the hierarchical clustering analysis in macrophages and liver are shown in (**A** and **B**) respectively, each column represents one sample, and each row represents one lncRNA probe set, the bar colors under the dendrogram represent the sample type: deep blue, control; light blue, case. The up-regulated (Red) and down-regulated (Green) genes were identified with three pre-set conditions (false discovery rate (FDR) < 20%, unfolded change *≥*2 and *p*-value < 0.01). (**C** and **D**) represent the number of lncRNA annotated by the RefSeq and the Ensembl database in macrophage and liver respectively. The overlap region represents for lncRNAs shared by two databases. (**E**) shows the co-expressed lncRNAs between macrophage and liver. (**F**) represents the number of differentially expressed lncRNAs in two microarray datasets, 5 lncRNAs in the overlap region were shared by macrophage and liver.

Based on the NetAffx annotation of the probe sets and the database of RefSeq and Ensembl annotation of lncRNAs, 202 probe sets (corresponding to 136 lncRNAs genes) in GSE13005 were identified. Of these, 34 probe sets (21 genes) were annotated as lncRNA by both RefSeq and Ensembl database (Figure 2C), 93 probe sets (62 genes) were annotated only by the RefSeq database, and 75 probe sets (53 genes) were annotated only by the Ensembl database. Meaning while, 1364 probe sets (corresponding to 1113 lncRNA genes) in GSE30861 were identified, among which 137 probe sets (92 genes) were annotated as lncRNA by both the RefSeq and the Ensembl database (Figure 2D), 390 probe sets (238 genes) were annotated only by the RefSeq database, and 837 probe sets (783 genes) were annotated only by the Ensembl database. Besides, probe sets that were annotated by both databases but had controversial definitions were excluded from this study, and the screening results were shown in Supplementary Tables 1 and 2.

### Identification of differentially expressed and co-expressed lncRNAs

**Figure 3:**
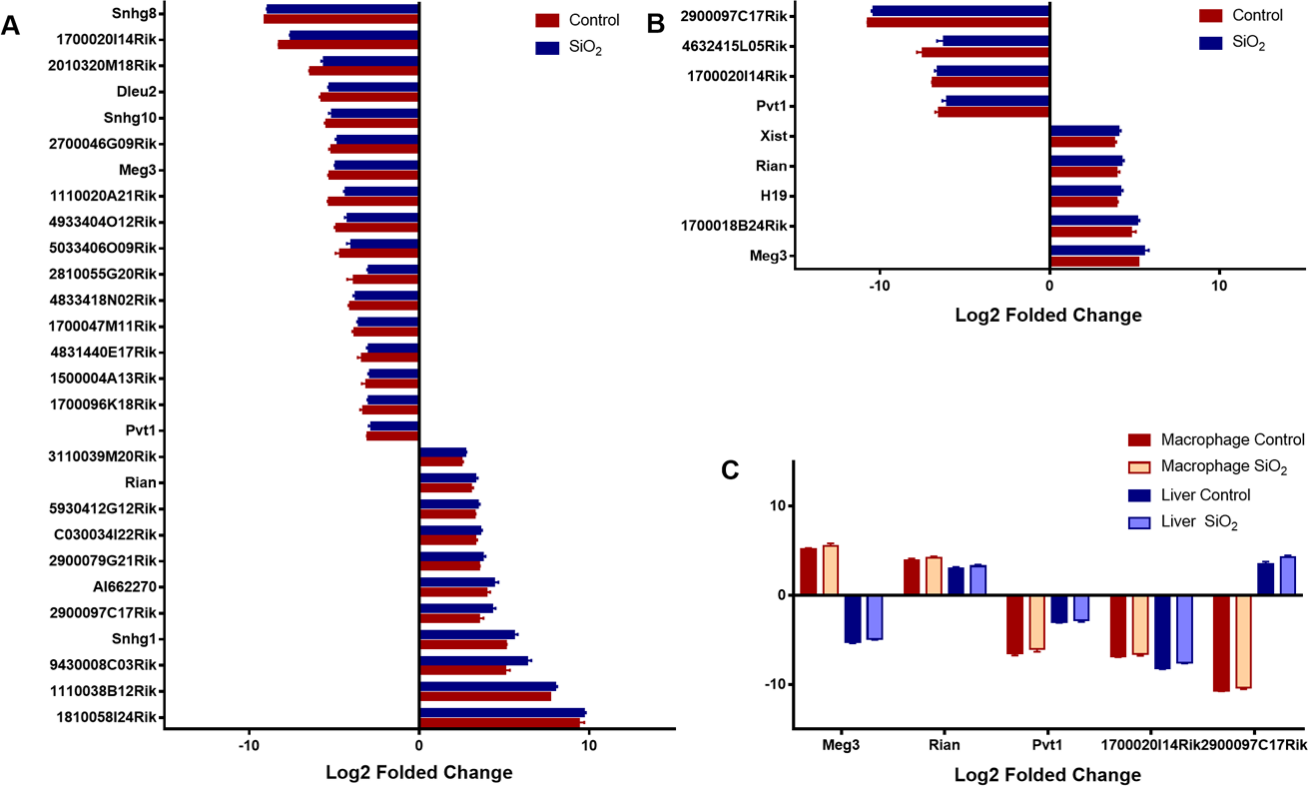
Differentially expressed lncRNAs The aberrantly expressed lncRNAs in liver and macrophage along with their corresponding controls were shown in (**A** and **B**) respectively (*p* < 0.01), lncRNAs both differentially expressed in macrophages and liver were shown in (**C**), the log_2_ folded change value was used to estimate the lncRNA expression levels before and after silica particle exposure.

Under conditions pre-set, nine differentially expressed lncRNAs in macrophages were identified (4 down-regulated and 5 up-regulated, Supplementary Table 3 and Figure 3B). As for liver, 28 differentially expressed lncRNAs were identified, among which 18 lncRNAs were down-regulated, and 10 were up-regulated (Supplementary Table 3 and Figure 3A). Additionally, lncRNAs such as Rain, Pvt1, Meg3, 2900097C17Rik, and 1700020I14Rik were all found differentially expressed both in macrophage and liver, besides, Rain, Pvt1 and 1700020I14Rik shown a similar regulatory trend, while Meg3 and 2900097C17Rik were oppositely regulated (Table 1, Figure 2E, 2F and Figure 3C). To further investigate the efficiency of the quality control process, all samples were clustered into two categories using HCA method, which correctly corresponded to the actual grouping method. The clustering results were shown in Figure 2A and 2B.

**Table 1:** Differently expressed lncRNAs shared by silica exposed macrophages and liver

No.	Probe set ID	Refseq transcript ID	Ensembl gene ID	Gene symbol	Gene title	Regulation (Macrophage/Liver)
1	1427580_a_at	NR_028261	ENSMUSG00000097451 ENSMUSG00000107391	Rian	RNA imprinted and accumulated in nucleus	UP/UP
2	1428055_at	NR_028261	ENSMUSG00000097451 ENSMUSG00000107391	Rian	RNA imprinted and accumulated in nucleus	UP/UP
3	1427140_at	NR_003368	ENSMUSG00000097039	Pvt1	plasmacytoma variant translocation 1	Down/Down
4	1452324_at	NR_003368	ENSMUSG00000097039	Pvt1	plasmacytoma variant translocation 1	Down/Down
5	1426758_s_at	NR_003633	ENSMUSG00000021268	Meg3	maternally expressed 3	UP/Down
6	1436057_at	NR_003633	ENSMUSG00000021268	Meg3	maternally expressed 3	UP/Down
7	1436713_s_at	NR_003633	ENSMUSG00000021268	Meg3	maternally expressed 3	UP/Down
8	1439380_x_at	NR_003633	ENSMUSG00000021268	Meg3	maternally expressed 3	UP/Down
9	1452183_a_at	NR_003633	ENSMUSG00000021268	Meg3	maternally expressed 3	UP/Down
10	1428286_at	NR_024329	ENSMUSG00000102869	2900097C17Rik	RIKEN cDNA 2900097C17 gene	Down/UP
11	1432646_a_at	NR_024329	ENSMUSG00000102869 ENSMUSG00000104222	2900097C17Rik	RIKEN cDNA 2900097C17 gene	Down/UP
12	1430989_a_at	NR_015473	ENSMUSG00000085438	1700020I14Rik	RIKEN cDNA 1700020I14 gene	Down/Down

### Enrichment analysis of nearest neighbor genes of differentially expressed lncRNAs

As a critical component of lncRNA regulatory mode, the potential *cis*-regulatory genes of the identified differentially expressed lncRNAs were predicted. Domains of 10 kb upstream or downstream of differentially expressed lncRNAs were investigated, 4 potential lncRNA targets including Rtl1, Z11981, Chp1, and Oip5 were proposed in this study (Table 2). To further illustrate their functions, GO analysis was adopted. We found that Oip5, Chp1, and Rtl1 were involved in 64 GO items, which mainly associated with multicellular organism development, cell division, cell cycle, protein transport, regulation of NF-κB transcription factor activity and membrane fusion (Table 3). The results suggested that one of the principal roles of lncRNAs may be transcriptional regulation of gene expression in cell proliferation and tissue inflammation (Supplementary Table 4).

**Table 2:** *cis* regulatory genes within 10 kbs up/down stream of differentially expressed lncRNAs

lncRNA	Chr	lncRNA Start	lncRNA End	Gene symbol	Gene Start	Gene End	Up/down stream	Gene title
Rian	chr12	109603945	109661711	Rtl1	109589192	109600330	Upstream	retrotransposon-like 1 (Rtl1)
Pvt1	chr15	62037986	62250976	Z11981	62176887	62180977	Overlap	M.musculus Pvt-1 mRNA
Meg3	chr12	109545398	109568650	NA	NA	NA	NA	NA
2900097C17Rik	chr2	156388063	156392979	NA	NA	NA	NA	NA
1700020I14Rik	chr2	119594296	119600744	Chp1	119547706	119587022	Upstream	calcineurin-like EF hand protein 1 (Chp1)
1700020I14Rik	chr2	119594296	119600744	Oip5	119609531	119618505	Downstream	Opa interacting protein 5 (Oip5)

*Note*: NA, not available.

**Table 3:** Functional enrichment terms shared by macrophages and liver

No.	Category	Term	Gene count		Fold enrichment score		Shared Genes
			Macrophage	Liver	Macrophage	Liver	
1	Biological Process	GO:0032570	3	5	8.78	4.26	NA
2	Biological Process	GO:0030890	4	6	8.17	3.56	MEF2C
3	Biological Process	GO:0006974	14	28	2.93	1.7	NA
4	Biological Process	GO:0042493	11	26	2.85	1.96	CROT
5	Biological Process	GO:0043065	9	29	2.36	2.21	GADD45B
6	Biological Process	GO:0006915	15	50	2.31	2.24	MEF2C, GADD45B, DRAM1
7	Biological Process	GO:0000122	18	41	2.17	1.44	MEF2C, MXD1
8	Biological Process	GO:0045944	22	66	1.94	1.69	MEF2C, NFATC3
9	Biological Process	GO:0007049	13	36	1.86	1.5	MIS12
10	Cellular Component	GO:0016605	5	9	4.55	2.48	NA
11	Cellular Component	GO:0000790	8	16	3.07	1.85	MXD1
12	Cellular Component	GO:0005789	16	45	1.97	1.67	FADS1, STX17, SRD5A3
13	Cellular Component	GO:0005783	25	64	1.65	1.28	TUSC3, FADS1, STX17, SRD5A3
14	Cellular Component	GO:0005737	99	340	1.3	1.36	MEF2C, SPATA13, STX17, LPP, SRD5A3, TOP2B, GADD45B, NFATC3, DRAM1
15	Molecular Function	GO:0001077	8	23	2.57	2.14	MEF2C, NFATC3
16	Molecular Function	GO:0000978	10	27	2.42	1.89	MEF2C, MXD1, NFATC3
17	Molecular Function	GO:0003682	11	32	2.05	1.72	MEF2C, TOP2B, NFATC3
18	Molecular Function	GO:0008270	22	58	1.78	1.35	LPP, ERI2
19	Molecular Function	GO:0003677	37	93	1.74	1.26	MEF2C, FBXO21, TOP2B, NFATC3, MXD1
20	Molecular Function	GO:0016740	26	75	1.53	1.28	CROT
21	Molecular Function	GO:0005515	70	242	1.48	1.48	MEF2C, MXD1, GADD45B, CSF1, ITM2B, NFATC3, LPP
22	KEGG Pathway	mmu05132	4	10	4.26	2.84	CXCL2
23	KEGG Pathway	mmu04380	5	14	3.29	2.46	CSF1
24	KEGG Pathway	mmu05164	6	25	2.91	3.23	NA
25	KEGG Pathway	mmu05166	9	21	2.69	1.67	NFATC3

*Note*: NA, not avaliable.

### Prediction of co-expressed genes of differentially expressed lncRNAs

**Figure 4:**
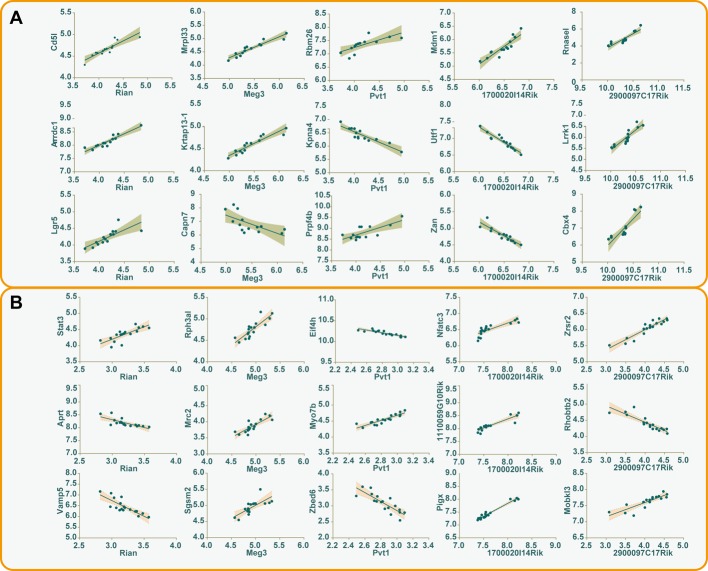
The correlations between differentially expressed lncRNAs and *trans*-regulatory genes The first three *trans*-regulatory genes along with their corresponding differentially expressed lncRNAs were used to construct correlative plots, and the 95% confidence intervals of every correlation were shown in the shadow of different colors (**A**) macrophage, gray; (**B**) liver, pink.

Co-expression analysis was adopted to explore the potential *trans*-regulatory targets of differentially expressed lncRNAs. Under the pre-set conditions (the correlation coefficient more than or equal to 0.8), a total number of 287 interaction relationships in silica stimulated macrophages were detected, including 150 positively correlated *trans*-regulatory genes and 117 negatively correlated *trans*-regulatory genes. While in liver microarrays, 852 interaction relationships were identified, 610 genes were positively correlated, and 242 genes were negatively correlated (Supplementary Table 5). The relationship between the first three *trans*-regulatory genes and every differentially expressed lncRNAs that shared by macrophage and liver was shown in Figure 4.

**Figure 5:**
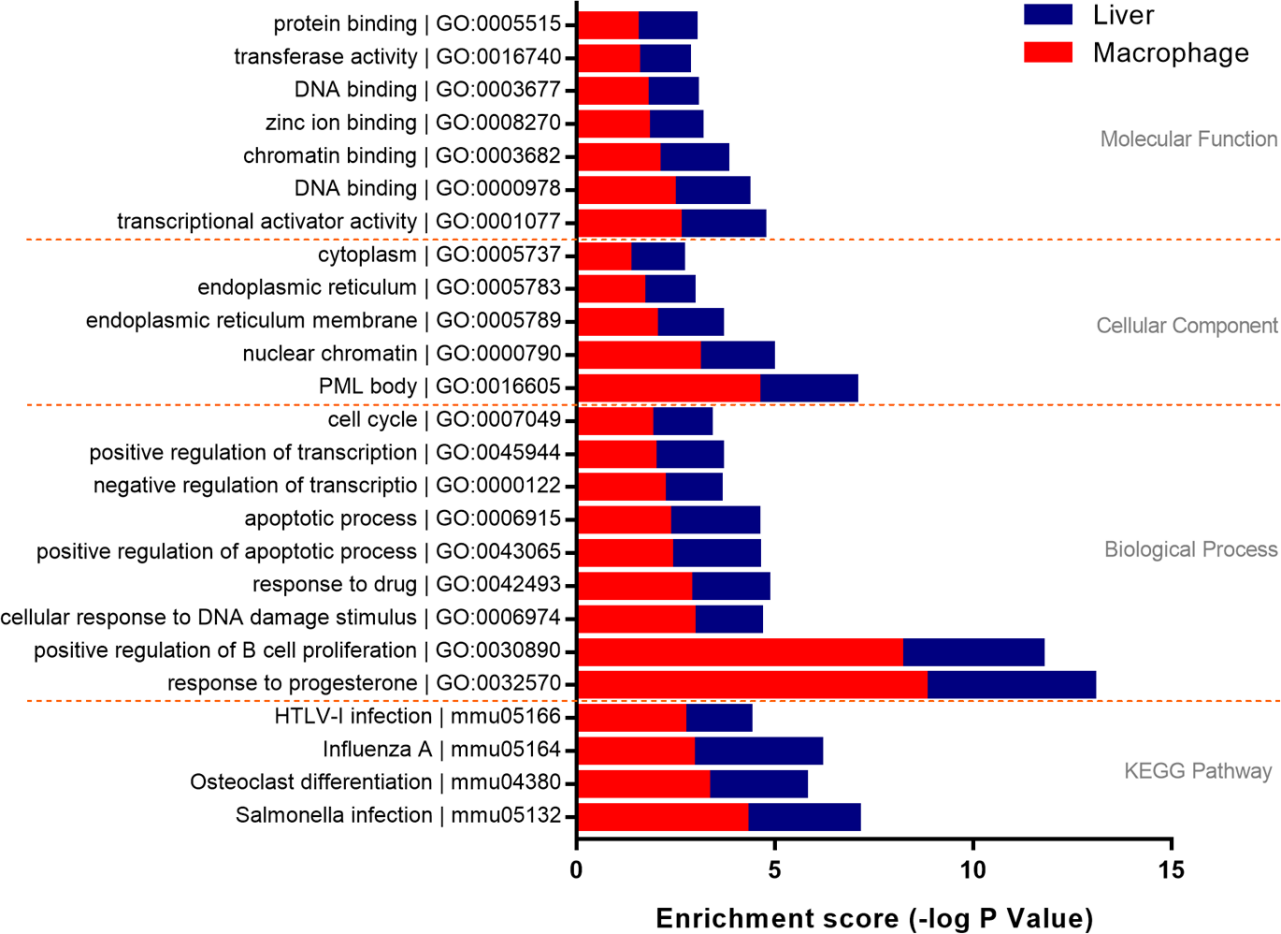
Functional enrichment analysis of *trans*-regulatory genes Genes with a Spearman correlation coefficient *≥*0.8 were involved in functional enrichment analysis. 21 GO items and 4 KEGG items shared by macrophage and liver were proposed. The enrichment score was used to evaluate the validity of every item, the length of bars both in macrophage (red) and liver (blue) gradually extended from first to last at every level separated by yellow dotted lines.

Results of functional enrichment analysis of the *trans*-regulatory genes indicated that the co-expressed genes in macrophages were enriched in 92 GO items, three aspects of which were included: biological process 56 items, cellular components 16 items, molecular function 20 items. Similarly, 362 GO items were enriched in liver probe sets (233 under biological process, 55 under cellular components, 74 under molecular function). In addition, 6 KEGG pathways were enriched in macrophages, and 60 items were proposed in liver. In order to further explore the role of macrophage in liver disorders, all GO items and KEGG pathways enriched in different microarrays were compared. As a result, 35 GO items were proposed, which mainly distributed in positive regulation of apoptotic process (GO:0043065), inflammatory response (GO:0006954) and immune system process (GO:0002376). 4 KEGG pathways including HTLV-I infection (mmu05166), Influenza A (mmu05164), Osteoclast differentiation (mmu04380) and Salmonella infection (mmu05132) were concluded (Supplementary Table 6, Figure 5). These findings suggested a critical role of macrophage in liver disorders that associated with silica particle exposure, and the biological processes and signaling pathways that included in silica particle stimulation were similar to virus infectious conditions.

### Co-acting proteins enrichment

**Figure 6:**
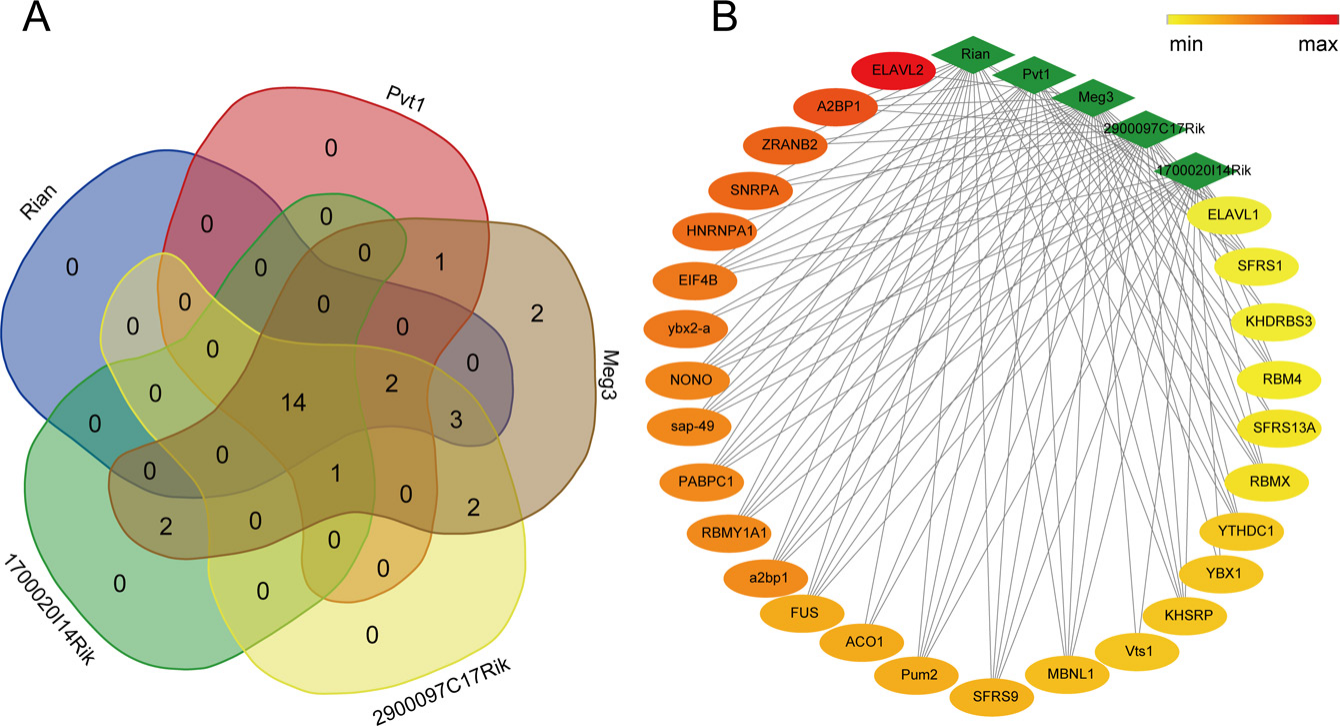
Interactions between differentially expressed lncRNAs and their co-acting proteins 14 co-acting proteins shared by lncRNA Rian, Pvt1, Meg3, 2900097C17Rik, and 1700020I14Rik were shown in (**A**). The network of the lncRNAs and their binding proteins (**B**) was constructed using the interaction score predicted by the RNA-Binding Protein DataBase, and the node color would change from red to yellow with the score decreasing.

The RBPDB is a collection of experimental observations of RNA-binding sites, both *in vitro* and *in vivo*, including 272 proteins as well as 71 binding profiles in the form of PWMs and sequence logos that extracted from a total of 1453 *in vitro* and *in vivo* experiments. To investigate the RNA-binding activity of proteins, the RBPDB was used to map and understand transcriptional and post-transcriptional networks and regulatory mechanisms of lncRNAs. In total, 27 proteins were identified via importing sequences of 5 differentially expressed lncRNAs into RBPDB respectively, of these, 14 proteins were shared by all differentially expressed lncRNAs (Supplementary Table 7, Figure 6). Results of functional enrichment analysis indicated that 8 proteins out of 9 in total including ELAVL1, KHSRP, RBMX, EIF4B, MBNL1, NONO, PABPC1, and Pum2 were associated with biological processes of mRNA splicing, regulation of translation and regulation of transcription, corresponding to the lncRNA regulation mode.

### Validation of differentially expressed lncRNAs

**Figure 7:**
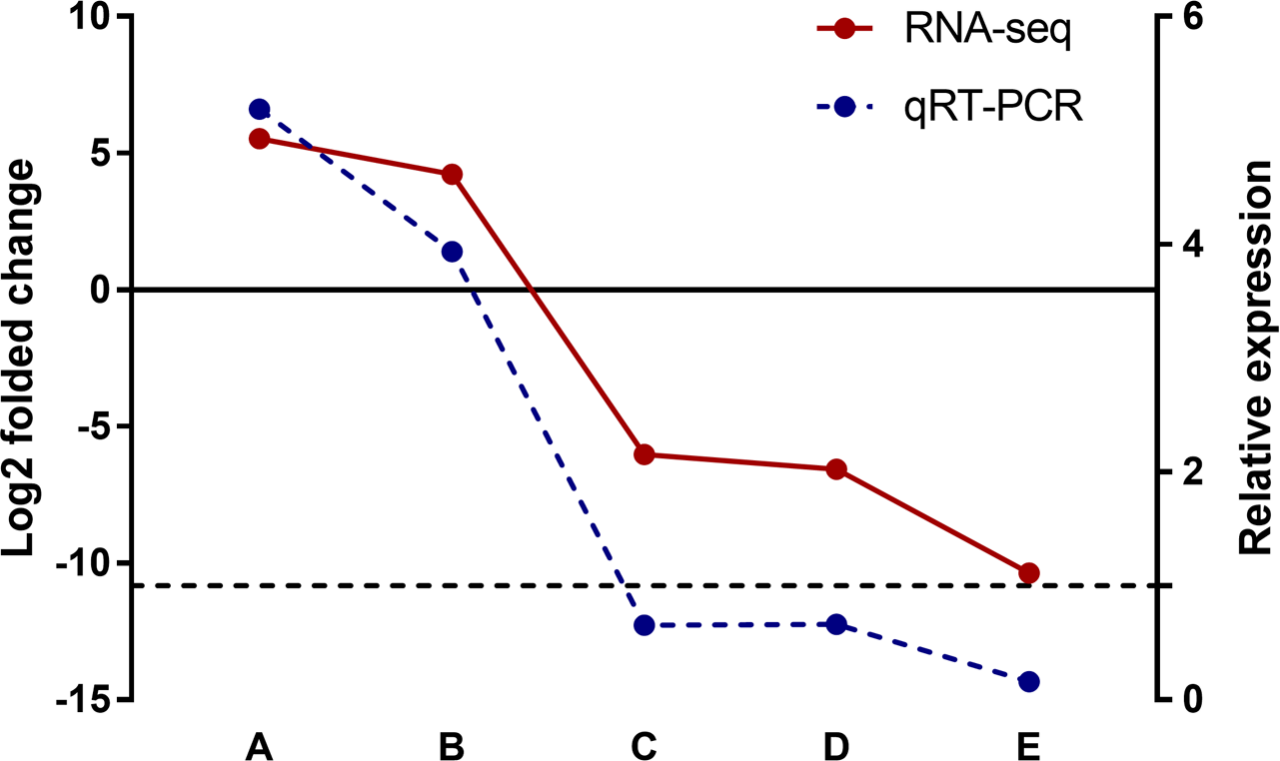
Validation of differentially expressed lncRNAs in silica particle exposed macrophages In GSE13005 microarray data set, the log_2_ folded change value of each differentially expressed lncRNA was shown in red color with a solid line, refering to the left Y axis. The up/down regulated lncRNAs were divied by the solid line (*Y* = 0), lncRNA Meg3 (**A**) and Rain (**B**) were up regulated (*p* < 0.01), while Pvt1 (**C**), 1700020I14Rik (**D**), and 2900097C17Rik (**E**) were down regulated (*p* < 0.01). Consistently, the lncRNAs involved in macrophages that exposed to a concentration of 100 μg/ml silica particles (3 samples in parallel per lncRNA) shown the same expression trend as RNA-Seq (the *p* valves of A–E were < 0.001, < 0.001, 0.002, 0.003, and < 0.001 respectively), and the relative expression levels of the selected lncRNAs were shown in blue color with a dotted line (right *Y* axis, *Y* = 1).

All dysregulated lncRNAs that shared by macrophage and liver were selected and examined for their expression patterns in silica particle stimulated macrophages. Consistent with the microarray datasets, the RT-PCR results confirmed that Meg3 and Rain were up regulated, while Pvt1, 2900097C17Rik, and 1700020I14Rik were down-regulated, suggesting that the identified lncRNAs were truly expressed, and the pipeline used in this study was highly strict in identifying putative lncRNAs (Figure 7).

## DISCUSSION

Over the past decades, lncRNAs have emerged as critical regulators in numerous diseases such as disorders in respiration, digestion, urination, hematopoiesis, and immunity [29]. Discoveries related to lncRNAs have initiated revolutionary progress in medical development, and significant developments have been made in profiling the molecular signatures of macrophage and liver using gene expression microarray datasets. As proved in many previous studies, there was extensive inflammatory cellular infiltration in dust exposed liver tissues, and macrophage has been validated as one of the most important initiators of inflammation [30], but roles of macrophage in liver diseases have not been fully investigated. Recently, hundreds of lncRNAs that altered expressions have been discovered in silica particle exposed macrophages and liver. Therefore, a new way to study the role of macrophage in liver disorders may emerge via profiling lncRNAs expressions in different microarray datasets, which may also contribute to identifying potential biomarkers for liver disease early diagnosis and treatment. In this study, to uncover the regulatory pattern of lncRNAs in macrophage and liver, we investigated the expression signatures of lncRNAs and analyzed their co-acting targets along with their functions, signaling pathways, and validated their expressions using RT-PCR.

With the development of biotechnologies, the next generation sequencing technology has been widely used in life scientific research. The GEO, a public gene expression data repository, offers abundant gene expression samples shared by previous studies. The Affymetrix Gene Chip was one of the most commonly used commercial microarrays in biological sample profiling. The Affymetrix Mouse Genome 430 2.0 array and its counterpart 430A that used in this study were re-annotated with NetAffx Annotation Files and the Ensembl database online. The probe-centric microarray datasets with the RefSeq and the Ensembl gene IDs were filtered by lncRNA extraction pipeline, which proved to be a feasible and attractive method with high accuracy and low cost, the advances of this pipeline also embody in practical reliability and simplicity compared with transcript sequencing analysis. As far as we know, this is the first time to investigate the role of macrophage in liver disorders via lncRNA sequencing.

### Overall changes of lncRNAs in macrophage and liver

In this study, we identified a set of differentially expressed lncRNAs in macrophage and liver (9 vs. 28), among which 5 lncRNAs were both identified in two microarray datasets. Compared with non-silica exposure groups, lncRNA Rain in the silica particle exposure group was up regulated, Pvt1 and 1700020I14Rik were down-regulated. While Meg3 and 2900097C17Rik were oppositely regulated between macrophage and liver. Such differentiations may be caused by biological processes in which they involved. On the other hand, the different expressions also signify potential roles of macrophage in liver diseases. Although a certain amount of lncRNAs have been reported to be aberrantly expressed in many diseases [31–33], studies related to those lncRNAs seldom concentrated on liver diseases. Pvt1, for example, has been identified as a candidate oncogene in humans, the overexpression of this gene was correlated with cancers of the breast, colorectum, ovary, and even hematological malignancies [34, 35]. Besides, Pvt1 has also been found to be essential for cardiomyocytes size maintenance and cardiac hypertrophy development [36]. In this study, Pvt1 was down regulated both in macrophage and liver, the value of log_2_ folded change in macrophage was −6.0, much lower than that in liver (−2.8), such significant expression difference indicated an important role of macrophage in inhibiting liver disorders. Different with Pvt1, current studies on Rain that knew as Ras interacting protein 1 (Rasip1) were focused on its roles in regulating vascular endothelial stability, and some studies indicated that it was essential for endothelial cell motility, angiogenesis and vessel formation [37, 38]. The much higher expression level of Rain in macrophage compared with liver (the value of log_2_ folded change: 4.2 vs. 3.3) suggested that liver vascular tubulogenesis and fibrosis may be induced by macrophage. Another candidate, lncRNA 1700020I14Rik that also named as Cyrano was seldom investigated, and the effect of this gene on embryonic development was only identified in zebrafish [39]. However, 1700020I14Rik showed a −6.6 and −7.5 folded change by log_2_ transformation in macrophage and liver respectively, which may be of great potential in predicting macrophage effect on liver diseases, thus, the function of 1700020I14Rik should be further studied. Another two candidates, lncRNA MEG3 and 2900097C17Rik that expressed conversely may explain the protective property of macrophage in the occurrence and development of liver disorders. Meg3 was found to be expressed in cancers and function as negative regulators of growth, cancer inhibitors [40, 41]. Consistently, it was up regulated with a log_2_ folded change value of 5.5 in macrophage but down regulated by −4.9 log_2_ folded change in liver. As for 2900097C17Rik, there are only a few number of available literatures that had recorded its existence but without further description and functional investigation [42]. In this study, 2900097C17Rik was down regulated by −10.4 log_2_ folded change in macrophage and up regulated by 4.3 log_2_ folded change in liver. Considering the functional pattern of Meg3, we may conclude that lncRNA 2900097C17Rik is an inhibitory regulator of inflammation in macrophage but up regulated in liver disease.

### Functional enrichment analysis of differentially expressed lncRNAs shared by macrophage and liver

In order to identify functional patterns and signaling pathways related to differentially expressed lncRNAs shared by macrophage and liver, we processed bioinformatics analysis including GO analysis, KEGG enrichment, protein-protein interaction network construction, and Spearman correlation analysis from three aspects of *cis* regulation, *trans* regulation, and co-acting protein complex regulation. Firstly, *cis* regulatory genes distributed in the domain of 10 kb upstream or downstream of differentially expressed lncRNAs were investigated. For example, Rtl1 that corresponded to lncRNA Rain was mainly involved in the biological process of cell cycle (GO: 0007049) and cell division (GO: 0051301). Oip5 and Chp1 that associated with lncRNA 1700020I14Rik participate in protein transportation (GO: 0015031), regulation of NF-kappaB transcription factor activity (GO: 0032088), and multicellular organism development (GO: 0007275). In consistent with previous studies, Rtl1 along with paternally expressed 11 (Peg11) were involved in the maintenance of fetal capillaries, it would cause fetal lethality or late fetal growth retardation in the absence of Rtl1/Peg11 [43], but the regulatory mechanisms are still not clear. Thus, the *cis* regulatory pattern such as Rain-Rtl1 that first concluded from this study may provide new sights on the explanation of vascular anomaly, which widely distributed in liver disorders. Moreover, Oip5 was previously proved to be associated with adipose proliferation and was found up regulated in obesity [44]. Chp1 was required for axon degeneration in many neurodegenerative diseases [45], and Chp2, the homologous gene of Chp1, could enhance the oncogenic potential of HEK293 cells through activating the calcineurin/nuclear factor signaling pathway in activated T cells [46]. Consequently, the occurrence and development of cirrhosis and hepatocellular carcinoma may be regulated by 1700020I14Rik- Oip5 and-Chp interactions.

Secondly, to elucidate potential mechanisms related to lncRNA-*trans* regulatory genes, a Spearman coefficient ≥0.8 was used to screen such genes. GO items shared by macrophage and liver were mainly involved in positive regulation of B cell proliferation (GO: 0030890), positive regulation of apoptotic process (GO: 0043065), and apoptotic process (GO: 0006915). The co-expressed genes such as Mef2c and Gadd45b were also identified. As described in other studies, the up regulation of Mef2c promotes invasion and metastasis of pancreatic ductal adenocarcinoma [47]. Moreover, Mef2c played double-edged roles in hepatocellular carcinoma, which could mediate VEGF-induced malignancy enhancement and also inhibit hepatoma carcinoma cells proliferation through blockade of Wnt/β-catenin signaling [48]. Additionally, results of the KEGG pathway enrichment indicated that signaling pathways involved in silica particle stimulation were similar to bacterial or viral infections. Accordingly, Mef2c and Gadd45b, corresponding to lncRNA 2900097C17Rik, may be involved in liver diseases in a bacterial or viral response-like manner.

Finally, we imported all sequences of differentially expressed lncRNAs into RBPDB to predict protein binding sites by PWMs. Protein MBNL1, which was co-acted by all differentially expressed lncRNAs, was found in human breast cancer and colorectal cancer [49]. In detail, MBNL1 could suppress cancer metastasis via binding to the 3′ untranslated regions (UTRs) of DBNL in breast and contribute to the carcinogenesis in the form of miRNA-MBNL1 in colorectum [50]. Other proteins such as ELAVL1, KHSRP, RBMX, EIF4B, NONO, PABPC1 and PUM2 were predicted to be transcriptional or post-transcriptional regulators. However, to our best knowledge, the protein-lncRNA interactions identified in this study have never been investigated.

In conclusion, we have identified and validated 5 differentially expressed lncRNAs that shared by silica particle exposed macrophages and liver in this study. Functional enrichment analysis of the aberrantly expressed lncRNAs suggested that macrophage plays important roles in the occurrence and development of liver diseases, the lncRNA regulatory patterns such as *cis*, *trans* regulatory genes and co-acting proteins were predicted to be of great potential in elucidating mechanisms of liver disorders. Besides, some linkages between lncRNAs and the potential regulatory genes or proteins were established for the first time, which would help to identify potential biomarkers and therapeutic targets in the diagnosis and prognosis of liver diseases, but further studies are still needed to provide evidence for the hypotheses that raised in this study in the future.

## MATERIALS AND METHODS

### GEO macrophage and liver gene expression data

Silica particle exposed macrophage and liver gene expression data were obtained from the GEO, which is publicly available. To compare the lncRNA expression signatures between macrophage and liver, two panels of macrophage and liver gene expression data sets were included in this study: GSE13005 and GSE30861. The raw files of these two data sets, which were based on the Affymetrix Mouse Genome 430A 2.0 platform and 430 2.0 platform, were downloaded from the GEO. Considering the lower expression level of lncRNAs when compared with protein-coding genes, the data quartile normalization, background adjustment, and summarization were processed using the Robust Multichip Average software (RMA, 1.2.0 In Development). The software provides more consistent estimates of fold-changes than other bioinformatics tools and has been shown to be an effective measurement tool for lncRNA profiling data [25]. In order to gain more reliable datasets, interquartile range and median of normalized unscaled standard error (NUSE) and relative log expression (RLE) were also used to process quality control, expression values that fell outside the control limits for both NUSE metrics and RLE metrics were removed from the downstream analysis. With this, a set of probe ID-centric gene expression values was obtained.

### lncRNA classification pipeline

To evaluate the lncRNA expressions in the probe ID-centric macrophage and liver gene expression datasets, we adopted the lncRNA classification pipeline which had been previously described to identify lncRNAs represented on the Affymetrix Genome array [26]. Briefly, the probe set IDs of the Affymetrix Mouse Genome 430A 2.0 and 430 2.0 platform were mapped to the NetAffx Annotation Files (Mouse430A 2.0 Annotations, CSV format, release 35, 10/07/14 and Mouse430 2.0 Annotations, CSV format, release 35, 10/7/14, available on the Affymetrix official website: http://www.affymetrix.com), then the annotated probe ID-centric gene expression datasets were extracted, and the new data set included the probe set ID, gene symbol, gene title, Ensembl gene ID, RefSeq transcript ID. Second, we only retained probes that labeled as “NR_” in the RefSeq transcripts IDs annotation column and “lincRNA,” “processed_transcript,” “macro_lncRNA” or “misc_RNA” in Ensembl gene IDs annotation column, labels reserved in this step indicates non-coding RNAs in the RefSeq database and the Ensembl database. Third, the probe sets obtained in step 2 were further filtered using “transcript type” item in Ensembl database, probe sets with annotations including pseudogenes, rRNAs, microRNAs or other short RNAs (tRNAs, snRNAs, and snoRNAs) were removed. Finally, 202 annotated lncRNA transcripts with corresponding Affymetrix probe IDs in macrophage were generated, while 1364 annotated lncRNA transcripts in liver were extracted.

### Differentially expressed lncRNAs screening and functional enrichment

Gene-e software was used to determine the differentially expressed lncRNAs between the silica exposure group and the control group. The hierarchical clustering analysis (HCA) was performed among samples or lncRNAs with the average linkage method respectively, and three conditions were set for differentially expressed lncRNAs screening: false discovery rate (FDR) < 20%, unfolded change ≥ 2 and *p*-value < 0.01. Gene ontology (GO) analysis and Kyoto Encyclopedia of Genes and Genomes (KEGG) pathway enrichment were conducted under the pre-set conditions (thresholds account ≥ 2 and ease score £ 0.1) with DAVID 6.8 online (https://david.ncifcrf.gov/). For functional enrichment analysis, GO analysis was processed to organize genes into categories of biological process, cellular component, and molecular function, while KEGG pathway enrichment was used to identify corresponding signaling pathways of differentially expressed lncRNAs.

### Prediction of co-operational *cis-* or *trans*-regulatory gene targets and proteins

The coding genes that distributed in the domain of 10kb upstream or downstream of the identified differentially expressed lncRNAs were considered as potential *cis*-acting targets. The *trans*-acting genes were identified using co-expression analysis, the Spearman rank correlation coefficients between the differentially expressed lncRNAs and protein coding genes were calculated and used to determine potential *trans*-acting genes (absolute value ≥ 0.8). Moreover, genes predicted by *cis*- or *trans*-acting processes were used to form a gene list for functional enrichment analysis. Proteins involved in lncRNA-protein complexes were predicted by the RNA-Binding Protein DataBase (RBPDB), which was based on a wide variety of cellular processes including transcription, RNA splicing and processing, localization, stability and translation [27].

### Cell culture and silica exposure

Silica particles (purity ∼99%, particle size 0.5–10 μm) used to stimulate macrophages were produced by Sigma-Aldrich Co. LLC., USA. The murine macrophage cell line RAW264.7 cells were obtained from the National Infrastructure of Cell Line Resource, China. These cells were grown in DMEM medium with 10% fetal bovine serum (FBS) and a humidified air supplemented with 5% CO_2_ at 37°C. After culturing for three passages, the complete medium was replaced by FBS free DMEM medium or 100 μg/ml SiO_2_ containing FBS free DMEM medium, 24 hours later, cells were harvested and preserved at −80°C for further use.

### Real time PCR for differentially expressed lncRNAs validation

According to the manufacturer’s instruction, total RNA of macrophages was extracted and reverse-transcribed into cDNAs, the RNA concentration was measured using NanoDrop 2000 spectrophotometer (Thermo Fisher Scientific, Waltham, MA, USA), and the method used for cDNA amplification was used as previously described [28], the relative lncRNA expression level was calculated using ΔΔCt method. GADPH was selected as an endogenous reference transcript. All lncRNA expression values were normalized according to the GADPH expressions. Primers for differentially expressed lncRNAs were designed using Primer-BLAST online which is available at https://www.ncbi.nlm.nih.gov/tools/primer-blast and listed in Supplementary Table 8.

### Statistical analysis

All data were analyzed by SAS version 9.2 for windows (SAS Institute Inc., Cary, NC, USA). Continuous variables with normal distribution were shown as mean ± standard error of the mean (SEM). The correlation between the lncRNA expression and the protein coding gene were assessed by Spearman rank correlation analysis, and genes were considered statistically significant if the correlation *p* value less than 0.05. The position weight matrices (PWMs) and sequence logos with a relative score more than or equal to 80% were used to identify lncRNA binding proteins. Student’s *t*-test was used to compare lncRNA expressions before and after silica stimulation. A *p* value less than 0.05 was considered as statistically significant unless otherwise indicated.

## SUPPLEMENTARY MATERIALS FIGURES AND TABLES
















