# Supplementary material for: Long non-coding RNA deep sequencing reveals the role of macrophage in liver disorders
Source: Oncotarget. 2017 Dec 12;8(70):114966–79. doi: 10.18632/oncotarget.23154 (PMC5777746; doi:10.18632/oncotarget.23154)
Supplement: Supplementary file 1 [file oncotarget-08-114966-s001.pdf]

Long non-coding RNA deep sequencing reveals the role of macrophage in liver disorders

SUPPLEMENTARY MATERIALS

Supplementary Table 1: LncRNAs Represented on the Affymetrix Mouse Genome 430 2.0 Array Based on the Annotation of Refseq and Ensembl. See Supplementary_Table_1

Supplementary Table 2: lncRNAs Shared by Refseq and Ensembl Annotations in macrophage. See Supplementary_Table_2

Supplementary Table 3: Differently Expressed lncRNAs in macroghage. See Supplementary_Table_3

Supplementary Table 4: GO items related to cis regulatory genes

Catergary	Gene	Gene Title	GO items
GOTERM_BP_DIRECT	Oip5	Opa interacting protein 5(Oip5)	GO:0007049~cell cycle,GO:0007059~chromosome segregation,GO:0007067~mitotic nuclear division,GO:0034080~CENP-A containing nucleosome assembly,GO:0051301~cell division,
GOTERM_BP_DIRECT	Chp1	calcineurin-like EF hand protein 1(Chp1)	GO:0001578~microtubule bundle formation,GO:0001933~negative regulation of protein phosphorylation,GO:0006469~negative regulation of protein kinase activity,GO:0006611~protein export from nucleus,GO:0006810~transport,GO:0010923~negative regulation of phosphatase activity,GO:0015031~protein transport,GO:0017156~calcium ion regulated exocytosis,GO:0022406~membrane docking,GO:0031122~cytoplasmic microtubule organization,GO:0031397~negative regulation of protein ubiquitination,GO:0031953~negative regulation of protein autophosphorylation,GO:0032088~negative regulation of NF-kappaB transcription factor activity,GO:0032417~positive regulation of sodium:proton antiporter activity,GO:0042308~negative regulation of protein import into nucleus,GO:0045056~transcytosis,GO:0050821~protein stabilization,GO:0051222~positive regulation of protein transport,GO:0051259~protein oligomerization,GO:0051453~regulation of intracellular pH,GO:0060050~positive regulation of protein glycosylation,GO:0061024~membrane organization,GO:0061025~membrane fusion,GO:0070885~negative regulation of calcineurin-NFAT signaling cascade,GO:0071468~cellular response to acidic pH,GO:0090314~positive regulation of protein targeting to membrane,GO:1901214~regulation of neuron death,
GOTERM_BP_DIRECT	Rtl1	retrotransposon-like 1(Rtl1)	GO:0007275~multicellular organism development,
GOTERM_CC_DIRECT	Oip5	Opa interacting protein 5(Oip5)	GO:0000775~chromosome, centromeric region,GO:0000785~chromatin,GO:0005634~nucleus,GO:0005654~nucleoplasm,GO:0005694~chromosome,GO:0005737~cytoplasm,GO:0010369~chromocenter,GO:0015030~Cajal body,
GOTERM_CC_DIRECT	Chp1	calcineurin-like EF hand protein 1(Chp1)	GO:0000139~Golgi membrane,GO:0005634~nucleus,GO:0005737~cytoplasm,GO:0005783~endoplasmic reticulum,GO:0005793~endoplasmic reticulum-Golgi intermediate compartment,GO:0005829~cytosol,GO:0005856~cytoskeleton,GO:0005886~plasma membrane,GO:0005925~focal adhesion,GO:0015630~microtubule cytoskeleton,GO:0016020~membrane,GO:0030133~transport vesicle,GO:0070062~extracellular exosome,
GOTERM_CC_DIRECT	Rtl1	retrotransposon-like 1(Rtl1)	GO:0016020~membrane,GO:0016021~integral component of membrane,
GOTERM_MF_DIRECT	Oip5	Opa interacting protein 5(Oip5)	GO:0046872~metal ion binding,
GOTERM_MF_DIRECT	Chp1	calcineurin-like EF hand protein 1(Chp1)	GO:0004860~protein kinase inhibitor activity,GO:0005215~transporter activity,GO:0005509~calcium ion binding,GO:0008017~microtubule binding,GO:0019900~kinase binding,GO:0046872~metal ion binding,GO:0048306~calcium-dependent protein binding,

Supplementary Table 5: trans regulatory genes associated with differentially expressed lncRNAs in macrophage. See Supplementary_Table_5

Supplementary Table 6: Functional enrichment analysis of trans regulatory genes in macrophage. See Supplementary_Table_6

Supplementary Table 7: lncRNA Binding Proteins based on RBPDB database. See Supplementary_Table_7

Supplementary Table 8: Primers of differently expressed lncRNAs and GAPDH. See Supplementary_Table_8
